# Intra-Cranial Tumor

**Published:** 1883-06

**Authors:** William Pepper

**Affiliations:** Professor of Clinical Medicine in the University of Pennsylvania


BUFFALO

Medical and Surgical Journal.

Vol. XXII. JUNE, 1883. No. 11.

### Original Communications.

### Intra-Cranial Tumor.

A CLINICAL LECTURE DELIVERED AT THE HOSPITAL OF THE UNIVERSITY OF PENNSYLVANIA, JAN. 27, 1883.

By William Pepper, M. D., LL. D.,
Professor of Clinical Medicine in the University of Pennsylvania.

Reported by William H. Morrison, M. D.

Gentlemen—This patient has been sent to us for an opinion in regard to the nature of his trouble. As I have not yet examined him, I shall question him before you and will ask you to carefully note his replies.

He is thirty-eight years of age and has been engaged as a brakeman on the railroad; he had been previously a private watchman. The first evidence of disease was an attack which he had in October, 1880. He had gone to bed about eleven o'clock, and went to sleep. At about three o'clock, he was awakened by a severe pain; he felt a numbness throughout his body and could not move. Thinking it was a nightmare, he asked his bed-fellow to turn him over; he then lost the power of speaking, but still retained consciousness. The legs began to jerk and he felt a numbness beginning in the legs and extending gradually upwards until it passed out at the top of the head; he then lost consciousness. It took about half a minute for the numbness to pass from the legs to the head. The unconsciousness lasted, he thinks, about ten minutes. On coming to, he was very sleepy; he asked the friends who were around him what was the matter and went to sleep. He did not feel sore, and went to work as usual the next day. He had three other attacks exactly like this one; two of these occured in the morning before he had gotten out of bed, and one about ten o'clock in the morning. The second attack took place April 17th; the third, a week later, and the fourth, in the following June. In these attacks the eyes rolled and he bit his tongue.

Since then he has had attacks which have been limited to the left side and particularly to the left leg. These are accompanied by numbness, which gradually passes up the limb until it reaches the middle of the body, where it stops, twitching in the muscles of the leg and pain in the left testicle. These attacks sometimes affect the arm. The first of these occurred in June, 1881; since which time he has had a number, probably about ten, coming at intervals of from one to three months. They last for one or two minutes, but in the course of five minutes he is able to return to his work.

During all this time his general health continued good, his appetite and digestion were not impaired and the bowels were regular; there was no trouble with the kidneys; there was no fever, no night-sweats, no malaria, the weight kept up, and his mind was clear.

One day in July, 1882, he walked from his house to the depot, about one o'clock in the afternoon; the day was very hot and he did not take an umbrella. Having gotten to the depot, he walked through his train to the baggage car, but feeling some peculiar sensations, he walked back again and sat down in the last car. He then felt a numbness in the left side and had the sensation of pins and needles, particularly in the left arm and leg. The right side was not affected. He did not fall and did not lose consciousness. He was taken home, but the numbness and feeling of weight in the limb continued for a day and a half. Every once in a while, a wave would pass through the limb. This lasted but a few seconds. The left hand was a little clumsy. He could not tell where he was going to put it unless he looked.

Since July, he has had very severe headache. He had never had it before. It would come on when he went to work. Looking up, as he was lighting the lamps in the cars, seemed to induce it. It would increase in severity until he had gone to bed and would continue through the night; but after eating his breakfast it would disappear and not return until evening. About August 15th, the headache was so severe that it brought on an attack of vomiting, and since then he has been unable to to do any work. At first, the headache was limited to the top of the head; it now shifts its position and comes down over the brows and in the occipital region, and affects both sides.

The memory appears to be not impaired, although he thinks it is not as good as formerly. The hearing is acute. Noises disturb him. There is undue acuteness of hearing. With this, there is a sound on both sides as though something were bursting out through the ears; this seems to keep time with the heart beat, and is worse on lying down. There is then pulsatile tinnitus. During the past two months, there has been failure of eyesight. The vision in both eyes is almost lost, although light is noticed better with the right eye than with the left. He cannot read the largest print, and cannot recognize a person standing before him in a bright light. There is no ptosis. The orbicularis is perfect in its action. The pupils are equal and about normal in size, with a little tendency to dilatation, but there is very little motion on the sudden admission of light after it has been excluded; in other words, the retina is not sensitive.

There is no twitching of the muscles in walking. There is a little giddiness on rising. He can stand as steadily when the eyes are shut as when they are open.

The heart's action is rapid, but entirely free from murmur.

He has been married since December, 1881; has had one child, who died at the age of seven months from "infantile convulsions." Has not been a drinking man; has never had any venereal trouble, and has never received any injury to the head; but four years ago while sitting in a wagon, the horse suddenly started and threw him out at the back. He fell on his shoulders and was stunned for a few moments. In a week he had recovered from the fall. He had no headache at this time. This is the only fall that he has had. (The patient was now removed.)

This is, unquestionably, a case of intra-cranial tumor, non-specific in character, and, as far as can be made out, with no definite connection with any injury. There is, without question, probably near the base of the brain, some growth. The symptoms are too characteristic to admit of doubt.

The diagnosis of intra-cranial tumor is at times excessively difficult; at other times, it is one of the easiest of all diagnoses. That which would lead you to suspect the existence of an intra-cranial tumor, I do not say that on which you would base a positive opinion, but that which would make you suspect the possibility of its existence, would be one of the following, or a group of the following symptoms: Very frequent, causeless vomiting without much nausea; obstinate and severe headache, without apparent cause and not yielding to ordinary remedies, especially if the headache be localized; giddiness on sudden change of position, and in progression, with perhaps some degree of unsteadiness in the gait; convulsive seizures, which may be genuine, general eclampsia, or what is more characteristic, unilateral spasms without loss of consciousness; paralysis of single cranial nerves, and progressive optic neuritis, with failure of vision. Either of these symptoms, if existing without apparent cause and resisting ordinary remedies, should arouse a suspicion of intra-cranial tumor. occasionally you will meet with cases where but one of these symptoms is present; for instance, I saw, a few years ago, in consultation, a young lady who had been absent from home and had been taken with obstinate vomiting, presumably due to some indiscretion in diet. The vomiting continued despite all treatment. It was rather a regurgitation of what had been taken and of the secretions of the stomach than an actual emesis; there was but little nausea. Some blood was brought up from time to time. Gastric ulcer was finally suspected, but it resisted all treatment for that condition. There was no headache complained of, nor was there any convulsive or spasmodic seizures. The vision remained normal, but later, giddiness and staggering in the gait came on. Consciousness was retained until lost from sheer inanition. At the post mortem, a sarcoma as large as a walnut was found situated in one lobe of the cerebellum. I have seen several cases in which persistent, uncontrollable vomiting, sometimes associated with optic neuritis and giddiness and sometimes without these, was the chief symptom of a tumor of the cerebellum or of the base of the brain.

There will sometimes be convulsive seizures, and especially of the Jacksonian type. These are unilateral or monoplegic spasmodic attacks, without loss of consciousness, and are extremely significant of localized coarse lesions within the cranium. They were admirably described by Hughlings Jackson, and have hence been called Jacksonian epilepsy. This man has had both kinds. He had four of general eclampsia, with loss of consciousness, rolling of the eyes, biting of the tongue and general spasmodic movements, just as in a case of epilepsy. Instead of these continuing and growing more severe, they have been substituted by a totally different sort of spasmodic attack. The latter have been unilateral, limited to the left side and affecting sometimes the leg and sometimes the arm. These are very characteristic. They will be found in cases of syphilitic nodes, gummata of the membranes of the brain and in localized specific pachymeningitis, when the degree of thickening is sufficient to cause pressure on the cortex. They are found in small growths on or near the surface of the brain, involving a certain convolution, and according to the convolution implicated, will be the locality of the spasm; so that not only can the nature of the trouble be diagnosed, but occasionally the position of the tumor may be determined. In the present case, it is difficult to define the precise location of the lesion, for, as you see, the spasms are not limited to one set of muscles; sometimes they affect the arms, and sometimes the legs. My opinion is that the lesion is rather coarse and extensive.

Pain in the head is one of the most constant symptoms of intra-cranial tumor. This pain is often agonizing, or as this patient describes it, "head-splitting." As you know the pains occurring as a result of specific disease are worse at night, but I have noted in many cases of intra-cranial tumor, even where there was no suspicion of specific taint, that the pain was chiefly nocturnal. The pain may, however, continue day and night. It may be violently paroxysmal, the paroxysms occurring at any time, but frequently at night. It may be strictly localized and bear a relation to the seat of the growth. It is at times compared to the driving of a nail into the brain. It may extend over certain regions, or it may involve the whole head.

Giddiness is frequently met with. It depends upon irritation of the cortex, irregular radiations of motor power thus resulting. There may be more than giddiness; ataxia may be actually present. The gait may not only be staggering, but resemble that of a patient with advanced posterior sclerosis.

Nothing is more characteristic when present, than paralysis of certain cranial nerves, as ptosis of one side. When associated with some of the other symptoms which I have mentioned this is very significant. Local palsies are of course most common when the tumor is on the under surface of the anterior portion of the brain, so as to press upon the nerve after its emergence from the cranial mass. Facial paralysis is not rarely met with.

Lastly, inflammation of the optic nerve, descending neuritis, has since the introduction of the opthalmoscope become an important sign in the diagnosis of intra-cranial tumors. Progressive optic neuritis occurs in a number of conditions. It may be present in chronic meningitis. It may exist as an independent affection in constitutional syphilis. Hence its existence, while a good corroborative evidence where there are other symptoms of intra-cranial tumor, is not of itself sufficient to justify the diagnosis; nor, on the other hand, is its absence sufficient to warrant the exclusion of the idea of intra-cranial tumor. In several cases of this kind under my care, repeated examinations of the eye-ground revealed no evidences of optic neuritis. The occurrence of optic neuritis depends to a great extent upon the position of the tumor. In certain situations, a tumor would neither interfere with the vascularity of the retina nor set up inflammation of the nerve. Even when optic neuritis is present, the vision may still retain considerable acuteness. The fact that the patient is able to see distant objects, does not suffice to exclude the existence of optic neuritis, for vision may not fail until late. An opthalmoscopic examination must always be made.

In this patient a considerable number of the symptoms which I have mentioned are present. There has been violent headache, shifting its location, eclampsia, unilateral spasmodic attacks, without loss of consciousness, giddiness in walking and on suddenly changing the position, progressive failure of vision until the man is partially blind, and exaggerated acuteness of hearing. Only once has there been vomiting, and that occurred after an unusually severe attack of blinding headache. We have not yet had an opportunity of having the eye-ground examined, but that will be done. We shall doubtless find advanced retinal and optic nerve changes.

The nature of this growth is of course uncertain. It may be said that it is not specific, for the man's character and record are good and his statements are clear on this point; nor is it the result of traumatism. The fall from the wagon did not implicate the cranial bones and this injury can scarcely be set up as a sufficient explanation of the intra-cranial trouble.

The character of growths which form in the skull are various. Aneurism should always be thought of. In certain cases, the symptoms are so characteristic that you may be able to diagnose that the tumor is an aneurism. The vessel most frequently involved is one of the middle cerebral arteries. You would suspect that the tumor was an aneurism from the co-existence of marked disease of the vessels with some tortuosity and dilatation of the radials and temporals, slight bulging of the arch of the aorta and resistance in the carotid trunks, with the progressive evidences of tumor with paralysis of the facial trunk, which in aneurism of the middle cerebral artery would be likely to be pressed upon. On several occasions, from consideration of these points and the locality of the spasms, I have been able to diagnose a small aneurism of the middle cerebral artery. In rare instances, the tumor is a hydatid cyst. The presence of retinal echinococci may enable the diagnosis to be made. In cases of intra-cranial tubercular tumors, tubercles may be found in the retina. A history of syphilis would indicate that the growth was specific. More frequently we can only say that there is a coarse lesion, a tumor, marked thickening of the membranes of the brain, or an exostosis on the bone.

In this patient I considered that the growth is on the right side of the brain, that it is near the base, and that it is somewhat toward the anterior portion of the cranium. As to its character, I have nothing to offer, but I believe it to be a solid growth.

As to the prognosis you can judge for yourselves. There is no reason to prescribe specific treatment. There is no reason to trephine, with the hope of removing an exostosis or coming upon a sub-periosteal abscess. The treatment is simply palliative, and is to be directed to relieving the headache and regulating the functions, but without any expectation of permanently influencing the progress of the disease. It is a distressing thing to have to formulate so fatal a prognosis, but I do not see how we can form any other opinion.

When I again meet you, I shall give you the results of the examination of the eye-ground.

The following is the report of the ophthalmoscopic examination made in the above case by Dr. S. D. Risley:

Right eye. Margins of nerve entirely hidden; location only seen by converging vessels. Macula apparently emmetropic. Upper part of disc most prominent. Upper temporal vein seen best with + 4.5 D. Numerous minute extravasations on the surface of the disc, but none elsewhere. White striations radiate from the nerve above and below. The veins are full and tortuous. The arteries are small and often missed from emptiness or from being lost in the retina. The upper temporal vein curves down and dips into the retina and is hidden from view one-half disc-diameter from the border.

Left eye. Upper temporal vein +4 D. Extravasations on nerve surface; greater opacity, the whole presenting a whiter and more striated appearance. Veins less full than on the other side. The arteries are even smaller and more empty and in many places dissappeared from view. Macular region apparently emmetropic. No extravasations except on disc and these absorbing. Neuritis probably commencing. No retrograde changes.

